# Anxiety-related gut microbiota alterations in Parkinson’s disease: distinct associations compared to healthy individuals

**DOI:** 10.3389/fcimb.2025.1594152

**Published:** 2025-06-18

**Authors:** Sheng-Hsuan Lin, Ru-Jen Lin, Kai-Yu Chan, Chia-Ling Chu, Yan-Lin Chen, Shih-Chen Fu

**Affiliations:** ^1^ Institute of Statistics, National Yang Ming Chiao Tung University, Hsinchu, Taiwan; ^2^ Institute of Data Science and Engineering, National Yang Ming Chiao Tung University, Hsinchu, Taiwan; ^3^ Department of Applied Mathematics, National Dong Hwa University, Hualien, Taiwan; ^4^ Department of Biochemistry and Molecular Medicine, National Dong Hwa University, Hualien, Taiwan; ^5^ Department of Neurology, National Taiwan University Hospital Hsin-Chu Branch, Hsinchu, Taiwan; ^6^ Graduate Institute of Genomics and Bioinformatics, National Chung Hsing University, Taichung, Taiwan

**Keywords:** gut microbiota, Parkinson’s disease, anxiety, microbial diversity, functional pathways, 16S rRNA sequencing

## Abstract

**Background and objectives:**

Anxiety affects 25–49% of Parkinson’s disease (PD) patients, exacerbating non-motor symptoms and significantly reducing quality of life. Growing evidence suggests that gut microbiota plays a role in anxiety, but whether its impact differs between PD and non-PD populations remains unclear. This study explores the heterogeneity of gut microbiota-associated anxiety in PD and non-PD individuals.

**Methods:**

Participants from the NeuroGenetics Research Consortium provided clinical data, including PD status, anxiety status, and stool samples analyzed via 16S rRNA sequencing. After excluding nine participants with missing anxiety data, 322 individuals were included (193 PD, 129 non-PD). We assessed α-diversity, β-diversity, taxonomic composition, and functional pathways to compare microbial differences between anxious and non-anxious individuals within and across PD and non-PD groups.

**Results:**

Beta diversity analysis revealed significant microbial differences between anxious and non-anxious PD patients (p = 0.043 in Bray-Curtis index) but not in the non-PD group. *Escherichia-Shigella* was significantly enriched in non-anxious PD patients (p = 0.011). Functional pathway analysis identified distinct metabolic alterations associated with anxiety in PD and non-PD individuals. In non-PD participants, anxiety was linked to increased activity in glycosphingolipid biosynthesis, sphingolipid metabolism, other glycan degradation, glycosphingolipid biosynthesis, and glycosaminoglycan degradation. In contrast, PD patients with anxiety exhibited enrichment in indole alkaloid biosynthesis, linoleic acid metabolism, and polyketide sugar unit biosynthesis.

**Conclusion:**

Gut microbiota-associated anxiety differs between PD and non-PD populations, suggesting distinct pathophysiological mechanisms. These findings underscore the potential of microbiome-targeted interventions as novel therapeutic strategies for anxiety in PD patients.

## Introduction

Parkinson’s disease (PD) is the second most common neurodegenerative disorder, with a global prevalence of 0.3% among individuals over 40 years of age (Pringsheim et al., 2014). PD is characterized by both motor and non-motor symptoms, with anxiety being one of the most prevalent and debilitating non-motor symptoms (Broen et al., 2016). Among PD patients, anxiety has an average prevalence of 31%, with non-episodic anxiety being more common than episodic forms (Broen et al., 2016). The most frequently diagnosed anxiety disorder is generalized anxiety disorder (GAD) (14.1%), followed by social anxiety disorder (13.8%) and panic disorder (6.8%) (Broen et al., 2016). Additionally, approximately one-third of PD patients meet the criteria for multiple anxiety disorders. Anxiety worsens movement difficulties, including sudden freezing while walking, and can also lead to cognitive issues, making daily life more challenging and reducing overall well-being (Broen et al., 2016).

Given its high prevalence and severe consequences, understanding the mechanisms underlying anxiety in PD is crucial for developing targeted interventions. Most existing studies on PD-related anxiety either attribute it to the physical discomfort caused by disease progression or focus on neuropathological factors such as dopaminergic and serotonergic dysfunction (Liu et al., 2020; Tanaka et al., 2023). However, these mechanisms do not fully explain why some PD patients develop severe anxiety while others do not, suggesting the involvement of additional pathophysiological factors.

Increasing evidence highlights the role of gut microbiota in neuropsychiatric disorders, including anxiety (Clapp et al., 2017; Li et al., 2018; Liu et al., 2020; Tanaka et al., 2023; MacKay et al., 2024). The gut-brain axis regulates neuroinflammation, stress responses, and neurotransmitter synthesis, which are all implicated in anxiety (Li et al., 2018; Liu et al., 2020; Tanaka et al., 2023). For example, MacKay et al. demonstrated that gut microbiota modulates brain plasticity, blood-brain barrier integrity, and neurotransmitter metabolism in different anxiety disorders (MacKay et al., 2024). Liu et al. (2020) reported that altering gut microbiota with antibiotics reduces anxiety symptoms (Liu et al., 2020), while Tanaka et al. (2023) found that *Bacteroides* abundance was linked to insomnia-related anxiety (Tanaka et al., 2023). Studies also suggest that gut dysbiosis is associated with non-motor symptoms such as reduced motivation, hallucinations and delusions in PD patients (Zhang et al., 2023). Despite these insights, it remains unclear whether gut microbiota influences anxiety differently in PD versus non-PD individuals, highlighting a critical gap in understanding the heterogeneity of the gut microbiota-anxiety relationship across different disease states.

Building on this background, this study aims to investigate whether gut microbiota composition and metabolic function differ between anxious and non-anxious PD individuals, compared to non-PD individuals. We hypothesize that gut microbiota plays a differential role in anxiety across PD and non-PD populations, leading to distinct microbial and metabolic pathway alterations in PD patients with anxiety.

## Methods

### Participant recruitment and data collection

We utilized data from the study by Hill-Burns et al (Hill-Burns et al., 2017), which originally included 376 participants enrolled in the NeuroGenetics Research Consortium between March 2014 and January 2015. Among which, 54 of the healthy controls were spouses of PD participants, which helped minimize differences in environmental exposure, lifestyle, and demographic factors. Detailed descriptions of the NeuroGenetics Research Consortium dataset, including its clinical and genetic characteristics, were previously provided by Hamza et al (Hamza et al., 2010). Following exclusions, 331 participants remained, comprising 200 individuals diagnosed with Parkinson’s disease (PD) based on the modified UK Brain Bank criteria (Clarke et al., 2016) (134 males, 66 females, mean age: 68.35 years) and 131 individuals who self-reported as being free from neurodegenerative conditions (52 males, 79 females, mean age: 70.37 years). Anxiety symptoms were assessed using a self-reported questionnaire. Additional exclusions included 9 participants with missing anxiety status. The final dataset for analysis comprised 322 participants: 193 PD patients (129 males, 64 females, mean age: 68.08 years) and 129 controls (52 males, 77 females, mean age: 70.22 years). Detailed procedures for fecal sample collection, DNA extraction, sequencing, and metadata collection can be found in Hill-Burns et al (Hill-Burns et al., 2017).

### Analysis of 16S rRNA sequence data

The 16S rRNA gene, a highly conserved bacterial marker, was targeted for sequencing to facilitate bacterial identification. Adapter sequences were trimmed using Trimmomatic v0.39 (Bolger et al., 2014). The subsequent reads were processed, aligned, and denoised using DADA2 v1.16 (Callahan et al., 2016). Specifically, sequence reads were filtered following the recommended DADA2 parameters, after which they were de-replicated and de-noised using default settings. An amplicon sequence variant (ASV) table was constructed, and taxonomy assignment was performed using the SILVA v132 database. Species-level annotations were assigned using the addSpecies function with SILVA as the reference. To standardize sequencing depth across samples, rarefaction was applied at a threshold of 5,000 reads per sample using the rarefy_even_depth function in phyloseq v1.32.0. Samples with fewer than 5,000 reads were removed before rarefaction. For subsequent statistical analyses, sequence counts were normalized to relative abundance by dividing the number of sequences assigned to each ASV by the total sequence count per sample. Only ASVs present in at least 10% of samples were retained for further analysis.

### Statistical analysis

Demographic characteristics—including age, sex, antibiotic or probiotic use, and dietary factors (fruits, vegetables, grains, meats, nuts, and yogurt)—were compared between individuals with and without anxiety within both the PD and control groups. The Kruskal-Wallis test was applied for continuous variables, while categorical variables were assessed using the chi-square test. To further address potential residual confounding, we have included age and sex as covariates for all downstream statistical analyses. To evaluate overall taxonomic diversity, we calculated both alpha and beta diversities between anxiety and non-anxiety groups. Alpha diversity metrics, including observed richness (ASV count), Chao1, Shannon, and Simpson indices (Chao, 1984; Rosenzweig, 1995; Magurran, 2013), were computed using phyloseq v1.50.0 (McMurdie and Holmes, 2013). P-values for alpha diversity were derived through analysis of variance with Stats 4.4.2. Beta diversity was assessed using unweighted UniFrac, weighted UniFrac (Lozupone et al., 2011), and Bray-Curtis distance (Bray and Curtis, 1957), all computed with phyloseq v1.50.0 (McMurdie and Holmes, 2013). ADONIS in the Vegan v2.6.4 package was used to calculate p-values for beta diversity. Additionally, microbial composition differences in relative abundance were analyzed separately for PD and control groups based on anxiety status. A generalized linear model incorporating a negative binomial distribution and a zero-inflated model was applied. False discovery rate (FDR) correction was applied to taxonomic analysis where a large number of individual comparisons were conducted. Whereas for alpha and beta diversity analyses, we did not apply additional multiple testing correction, consistent with common practice in microbiome research, particularly when only a small set of diversity metrics is tested and statistical power is limited.

### Functional enrichment analysis of predicted metagenomes

Metagenome functional composition was inferred using Phylogenetic Investigation of Communities by Reconstruction of Unobserved States (PICRUSt2) v2.4.1 (Douglas et al., 2020). The following pipeline was conducted, which included normalizing ASVs by copy number to account for variations in 16S rRNA gene copies across taxa, predicting functions based on Kyoto Encyclopedia of Genes and Genomes (KEGG) (Kanehisa and Goto, 2000) orthologs, and grouping predicted pathways according to KEGG hierarchical level 3. Within the PD and non-PD group, differences in metabolic pathways between individuals with and without anxiety were analyzed using the Statistical Analysis of Metagenomic Profiles (STAMP) software v2.1.3 (Parks et al., 2014). Comparisons were performed using White’s non-parametric t-test (two-sided, 1,000 replications), with statistical significance determined using a Storey false discovery rate threshold of <0.05.

### Data availability and ethical statement

The sequencing data utilized in this study are publicly available in the European Nucleotide Archive (ENA) under accession number ERP016332. All data are fully anonymized and contained no identifiable personal information. In accordance with standard ethical guidelines for secondary analyses of de-identified public datasets, ethical approval was not required for this study.

## Results

### Demography

A total of 331 participants were initially included in the study (200 PD patients, 131 healthy controls). After excluding nine individuals with missing anxiety data, the final cohort consisted of 322 participants (193 PD patients, 129 healthy controls), of whom 253 exhibited anxiety symptoms while 69 did not. We assessed and compared baseline characteristics between PD patients and healthy controls (see **Supplementary Table S1**). Among these variables, only sex showed a statistically significant difference between the groups. In the healthy control group, a significant association was observed between sex and anxiety status (p = 0.031), with a higher proportion of females in the anxious group (87.5%) compared to the non-anxious group (55.8%). However, no significant differences were found between anxious and non-anxious individuals in terms of age, antibiotic use, probiotic use, or dietary habits. Among PD patients, no significant differences were observed in sex distribution between the anxious and non-anxious groups. Similarly, age, antibiotic use, probiotic use, and dietary habits did not significantly differ between the two groups (**Table 1**). While age was not significantly associated with anxiety within either cohort, it remains a key risk factor for PD and may influence both anxiety prevalence and severity, as well as its relationship with gut microbiota. Therefore, all subsequent analyses were adjusted for age and sex to account for potential confounding effects. We also assessed disease duration in relation to anxiety among PD patients and found no significant differences between the groups, whether examined as a continuous variable or using a 10-year cutoff (**Table 1**; **Supplementary Figure S1**). In addition, we examined whether other microbiome-related variables - Levodopa use, constipation, and BMI - were associated with anxiety status. As shown in **Supplementary Table S2**, none of these variables were significantly associated with anxiety in either the PD or healthy control groups. This supports the decision not to include them as covariates in our final models, in order to preserve statistical power.

**Table 1:** Demographic characteristics of participants with and without anxiety in the PD and non-PD groups.

Sample characteristic	Non-Parkinson’s disease			Parkinson’s disease		
	Anxiety (N = 16)	w/o Anxiety (N = 113)	p-value	Anxiety (N = 53)	w/o Anxiety (N = 140)	p-value
Gender			0.031			0.179
Female	14 (87.5%)	63 (55.8%)		22 (41.5%)	42 (30.0%)	
Male	2 (12.5%)	50 (44.2%)		31 (58.5%)	98 (70.0%)	
Age			0.983			0.251
≦ 65	5 (31.2%)	31 (27.4%)		24 (45.3%)	49 (35.0%)	
> 65	11 (68.8%)	82 (72.6%)		29 (54.7%)	91 (65.0%)	
Antibiotics			0.747			0.520
No	16 (100.0%)	109 (96.5%)		51 (96.2%)	130 (92.9%)	
Yes	0 (0.0%)	3 (2.7%)		2 (3.8%)	7 (5.0%)	
Missing	0 (0.0%)	1 (0.9%)		0 (0.0%)	3 (2.1%)	
Probiotics			0.091			0.728
No	8 (50.0%)	85 (75.2%)		36 (67.9%)	103 (73.6%)	
Yes	7 (43.8%)	26 (23.0%)		13 (24.5%)	29 (20.7%)	
Missing	1 (6.2%)	2 (1.8%)		4 (7.5%)	8 (5.7%)	
Eat fruits or vegetable daily			0.764			0.544
No	1 (6.2%)	14 (12.4%)		9 (17.0%)	32 (22.9%)	
Yes	15 (93.8%)	99 (87.6%)		44 (83.0%)	107 (76.4%)	
Missing	0 (0.0%)	0 (0.0%)		0 (0.0%)	1 (0.7%)	
Eat grains daily			0.147			0.561
No	2 (12.5%)	40 (35.4%)		16 (30.2%)	42 (30.0%)	
Yes	14 (87.5%)	71 (62.8%)		37 (69.8%)	95 (67.9%)	
Missing	0 (0.0%)	2 (1.8%)		0 (0.0%)	3 (2.1%)	
Eat meats daily			0.393			0.649
No	8 (50.0%)	40 (35.4%)		24 (45.3%)	59 (42.1%)	
Yes	8 (50.0%)	73 (64.6%)		29 (54.7%)	79 (56.4%)	
Missing	0 (0.0%)	0 (0.0%)		0 (0.0%)	2 (1.4%)	
Eat nuts daily			0.306			0.544
No	9 (56.2%)	83 (73.5%)		44 (83.0%)	107 (76.4%)	
Yes	7 (43.8%)	29 (25.7%)		9 (17.0%)	32 (22.9%)	
Missing	0 (0.0%)	1 (0.9%)		0 (0.0%)	1 (0.7%)	
Eat yogurt daily			0.783			0.538
No	14 (87.5%)	101 (89.4%)		47 (88.7%)	129 (92.1%)	
Yes	2 (12.5%)	10 (8.8%)		6 (11.3%)	10 (7.1%)	
Missing	0 (0.0%)	2 (1.8%)		0 (0.0%)	1 (0.7%)	
Disease duration						0.165
≦ 10				23 (43.4%)	44 (31.4%)	
> 10				30 (56.6%)	96 (68.6%)	

### Effects of gut microbiota diversity on anxiety

**Figure 1:**
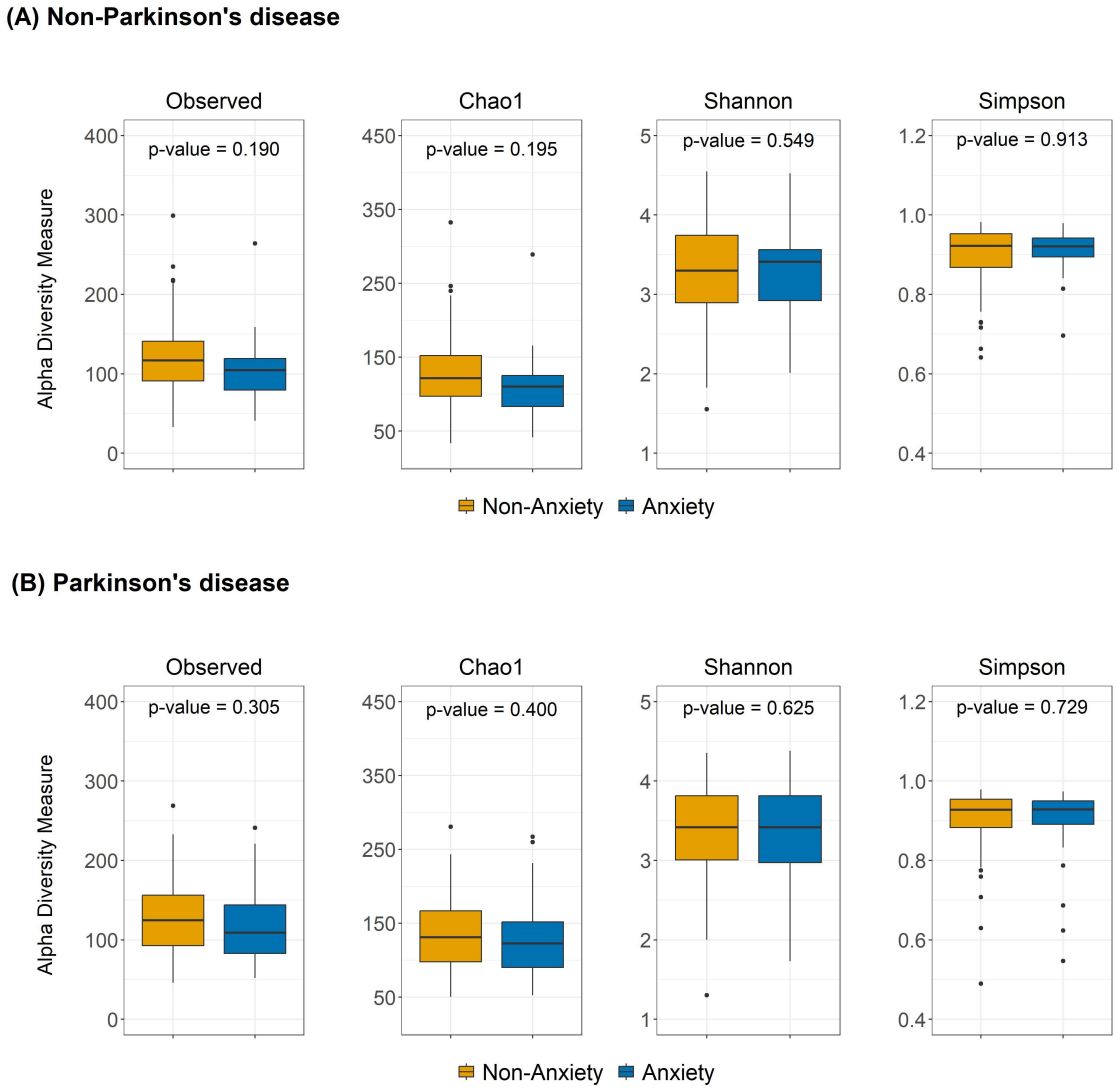
Alpha diversity differences between anxious and non-anxious individuals in both **(A)** non-PD and **(B)** PD groups. The boxplots show the alpha diversity of the bacterial communities measured by Observed ASVs, Chao1, Shannon, and Simpson indices. Median values, along with lower and upper quartiles, are displayed in the plots.

**Figure 2:**
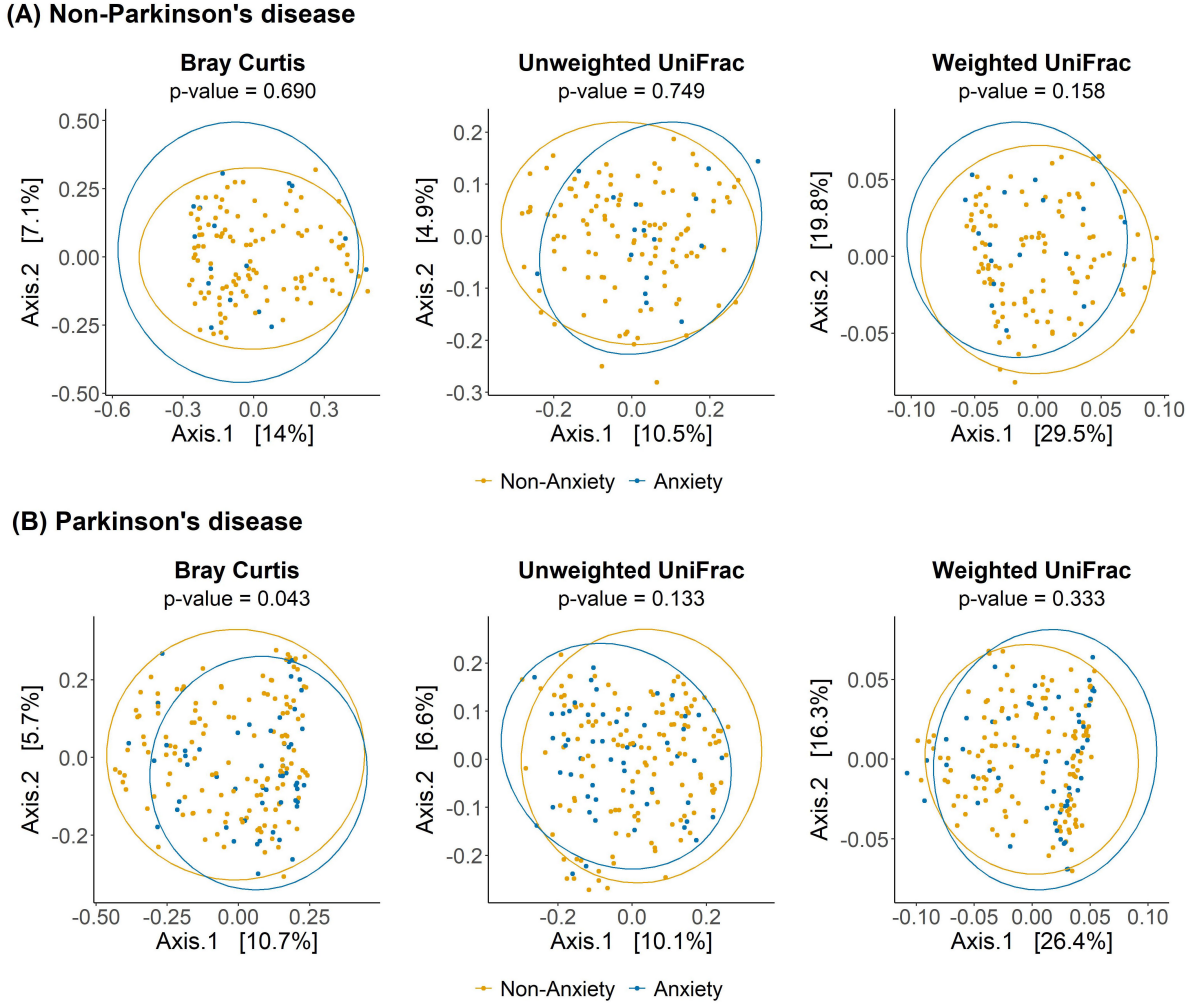
Beta diversity analysis of gut microbiota between anxious and non-anxious individuals in PD and non-PD groups. Principal coordinates analysis (PCoA) plots illustrate differences in microbial community composition between anxious and non-anxious individuals based on Bray-Curtis, Unweighted UniFrac, and Weighted UniFrac distance metrics.

As shown in **Figure 1**, no significant differences in alpha diversity were observed between anxious and non-anxious individuals in both the PD and non-PD groups. However, despite the lack of statistical significance, alpha diversity in the Observed and Chao1 indices tended to be lower in the anxiety group across both cohorts. In beta diversity analysis (**Figure 2**), no significant differences were detected in Bray-Curtis, Unweighted UniFrac, or Weighted UniFrac distance metrics among non-PD participants. In contrast, among PD patients, Bray-Curtis distance analysis revealed a significant group-level difference (p = 0.043), suggesting that the gut microbiota compositional structure might contribute to anxiety in PD patients. While the separation between groups was moderate, Axis 1 and Axis 2 explained 10.7% and 5.7% of the variance, respectively, indicating a discernible pattern of microbial variation associated with anxiety status in the PD cohort.

### Differences in gut microbiota relative abundance associated with anxiety

**Figure 3:**
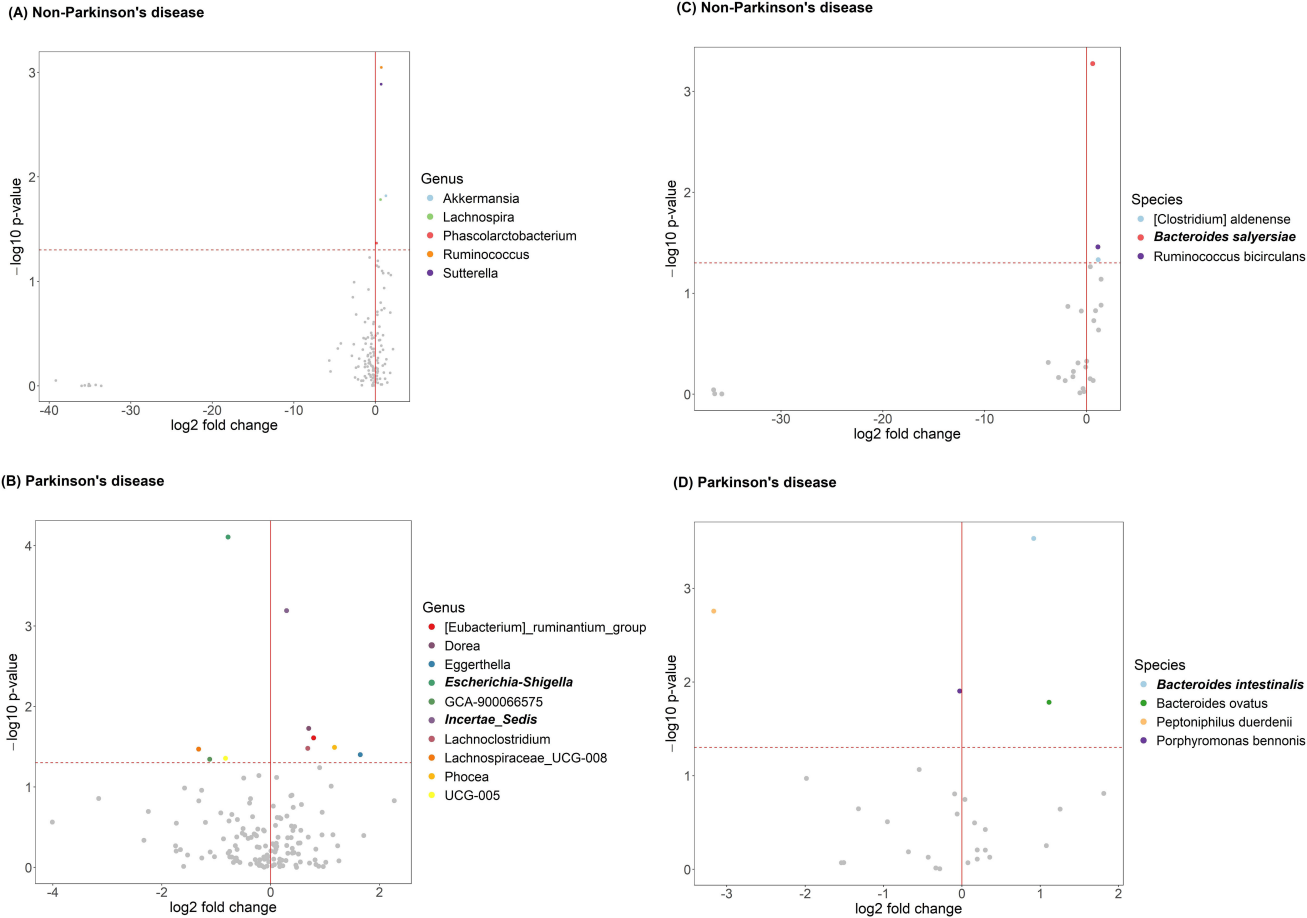
Anxiety-related gut microbiota in participants with and without Parkinson’s disease (PD), before and after FDR correction. **(A)** Differentially abundant genera in non-PD; **(B)** genera in PD; **(C)** species in non-PD; **(D)** species in PD. Each point represents a taxon, with log2 fold change (x-axis) and –log10 p-value (y-axis). The red vertical line marks no fold change; the horizontal dashed line marks the significance threshold. Bold labels indicate taxa significant after FDR correction. Colored points indicate taxa significant before FDR correction; gray points are non-significant.

**Figure 4:**
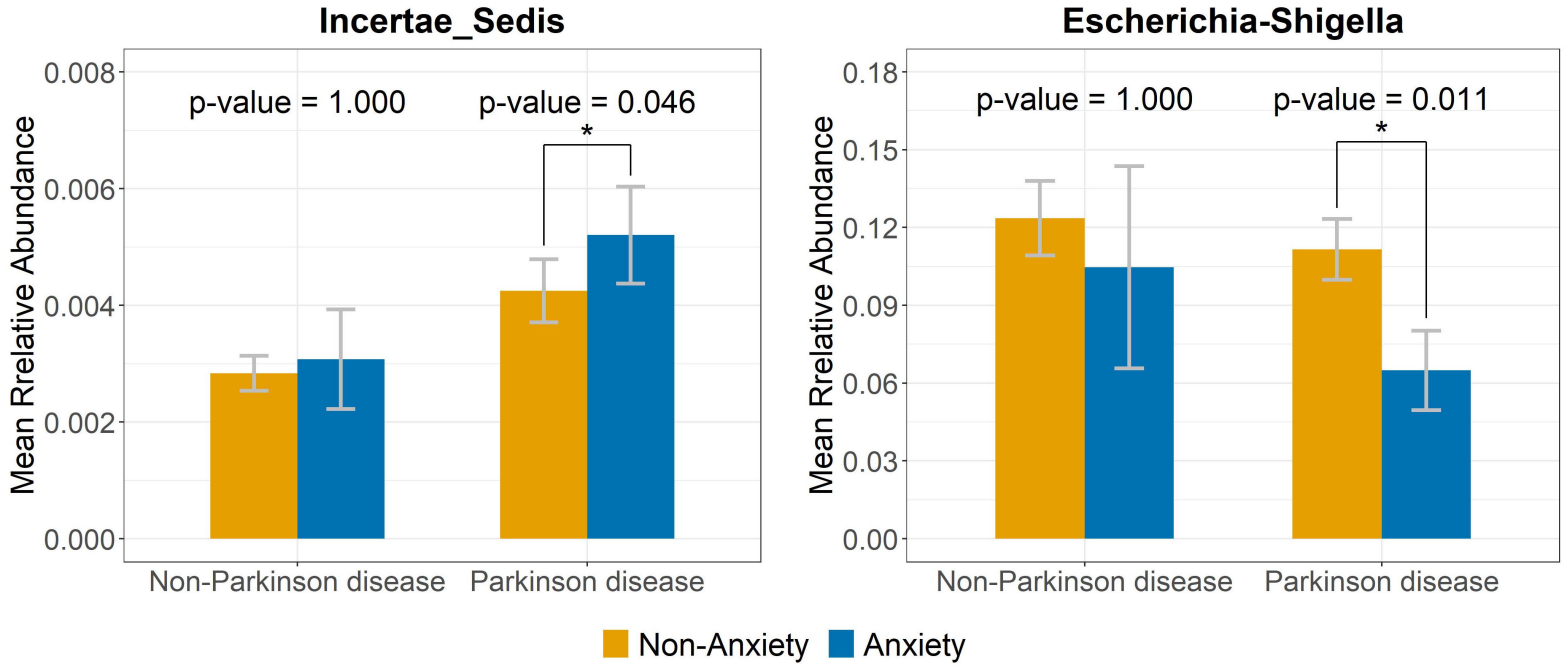
Relative abundance of bacterial genera linked to anxiety in PD but not in the non-PD group. Bar plots illustrate the relative abundance of *Incertae_Sedis* and *Escherichia-Shigella* in anxious and non-anxious PD and non-PD individuals. The contrasting abundance patterns suggest distinct microbiota signatures linked to anxiety in PD. Symbol * indicates that the genus shows a significant difference between the groups with and without anxiety.

The volcano plot illustrates significant differences in genus-level gut microbiota composition between anxious and non-anxious individuals (**Figures 3A, B**). Among non-PD participants, five genera initially showed significant differences; however, after FDR correction, no genera remained significant. In contrast, among PD patients, ten genera were significantly different before correction, and after correction, two taxa still showed significant differences. One belonged to the *Ruminococcaceae* family but remained taxonomically unclassified at the genus level (denoted as “*Incertae Sedis*” in the figure) and was significantly enriched in the anxious PD group (p = 0.046). Conversely, *Escherichia-Shigella* was significantly less abundant in the anxious PD group (p = 0.011). The relative abundance patterns of these two genera differed notably in the control group, shown in **Figure 4**.

At the species level (**Figures 3C, D**), distinct bacterial species were enriched in anxious individuals within each group. Among non-PD participants, three species initially showed significance before FDR correction; however, after correction, only *Bacteroides salyersiae* remained significantly enriched in the anxious group. Among PD patients, four species were significant before correction, but after FDR adjustment, only *Bacteroides intestinalis* remained significantly enriched in the anxious group, indicating a possible role in PD-related anxiety. These findings not only suggest a potential link between these species and anxiety symptoms but also indicate that the bacterial species associated with anxiety differ between PD and non-PD groups, highlighting potential disease-specific microbial signatures.

### Functional pathways associated with anxiety

**Figure 5:**
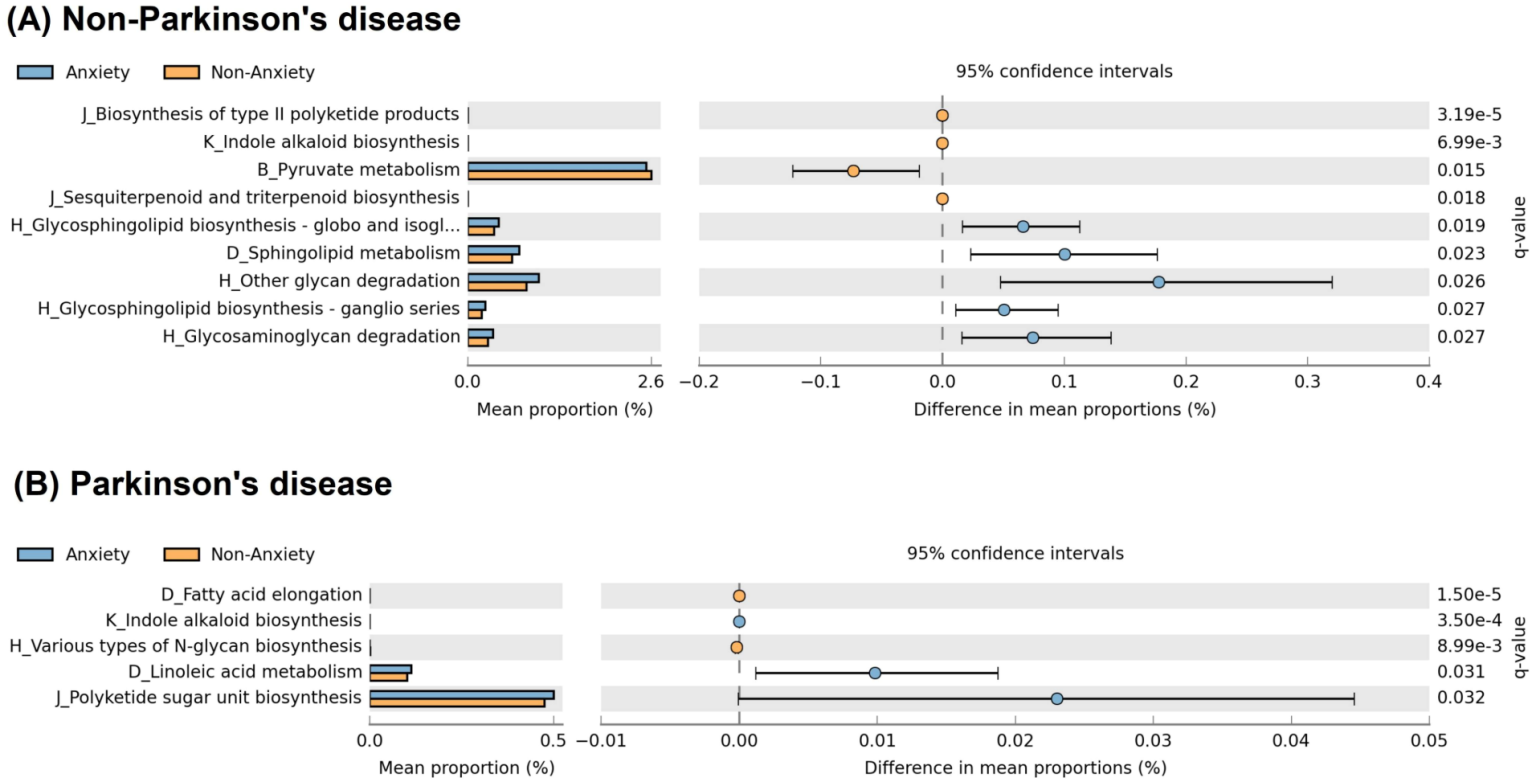
Functional pathway differences associated with anxiety in PD and non-PD groups. **(A)** In non-PD participants, anxiety was associated with increased glycosphingolipid biosynthesis, sphingolipid metabolism, and glycan degradation - pathways involved in membrane stability and neuroinflammation. **(B)** In PD patients, anxiety was linked to indole alkaloid biosynthesis, linoleic acid metabolism, and polyketide sugar unit biosynthesis, which may contribute to serotonergic dysregulation and neuroinflammatory processes. Pathway names on the y-axis follow KEGG hierarchy, shown as “Level 2 code_Level 3 classification,” where codes represent: B (carbohydrate metabolism), D (lipid metabolism), H (glycan biosynthesis and metabolism), J (metabolism of terpenoids and polyketides), and K (biosynthesis of other secondary metabolites). The full name for “H_Glycosphingolipid biosynthesis - globo and isogl…” in **(A)** is “H_Glycosphingolipid biosynthesis – globo and isoglobo series.”.

Analysis of functional pathways (**Figure 5**) revealed distinct metabolic alterations between anxious and non-anxious individuals in both PD and non-PD groups. Among non-PD participants, the anxious group exhibited significant enrichment in glycosphingolipid biosynthesis (globo and isoglobo series), sphingolipid metabolism, other glycan degradation, glycosphingolipid biosynthesis (ganglio series), and glycosaminoglycan degradation. In contrast, the non-anxious group showed higher activity in the biosynthesis of type II polyketide products, indole alkaloid biosynthesis, pyruvate metabolism, and sesquiterpenoid and triterpenoid biosynthesis. In PD patients, the pathways associated with anxiety differed entirely from those observed in the non-PD group. The anxious PD group showed significant enrichment in indole alkaloid biosynthesis, linoleic acid metabolism, and polyketide sugar unit biosynthesis, while the non-anxious PD group exhibited higher activity in fatty acid elongation and various types of N-glycan biosynthesis.

## Discussion

To our knowledge, this is the first study to investigate the relationship between anxiety and gut microbiota in PD patients, while also incorporating a non-PD comparison group to examine potential disease-specific heterogeneity. Our findings highlight that the association between gut microbiota and anxiety differs significantly between PD and non-PD individuals, suggesting that PD-related pathophysiology may uniquely shape gut microbiota-anxiety interactions. Although the original samples were collected between 2014 and 2015, we reanalyzed the data using updated microbiome processing pipelines - specifically, DADA2 and ASV-based methods - which offer higher taxonomic resolution and improved detection accuracy compared to traditional OTU-based approaches. This enhanced analytical framework strengthens the validity and relevance of our findings despite the age of the original samples.

One of the key findings of this study is that gut microbiota beta diversity was significantly altered in anxious PD patients, whereas no such differences were observed in non-PD participants, suggesting that microbial community composition may play a more pronounced role in anxiety within the PD population. Previous studies have generally supported an association between gut microbiota composition and anxiety symptoms, reporting that alpha diversity is typically lower in anxious individuals and that beta diversity differs between anxious and non-anxious groups (Li et al., 2018; Liu et al., 2020; MacKay et al., 2024). However, our results indicate that while alpha diversity in the anxious group tended to be lower specifically in the Observed and Chao1 indices across both PD and healthy populations, this difference did not reach statistical significance. Additionally, while prior research has demonstrated anxiety-related gut microbiota differences in general populations regardless of PD status (Tanaka et al., 2023), our study found that significant beta diversity differences were exclusive to PD patients, with no observable differences in the non-PD group. This finding suggests that in PD patients, gut microbiota compositional changes may play a more central role in the development of anxiety, highlighting the potential disease-specific nature of gut microbiota-anxiety interactions.

At the genus and species levels, our study further emphasizes that the relationship between anxiety and gut microbiota may differ depending on disease status. Previous research has suggested that probiotic genera such as *Lactobacillus* and *Bifidobacterium* may have anxiolytic effects (Li et al., 2018). However, in the non-PD group of our study, while several bacterial genera initially appeared to be significantly associated with anxiety before FDR correction, none remained significant after correction. Differences in gut microbiome findings across studies may also reflect variation of microbiome associations in anxiety subtypes. While Butler et al. (2023) reported elevated *Anaeromassilibacillus* sp *An250* in non-PD individuals with social anxiety disorder, our study - focused on individuals with generalized anxiety disorder - identified higher levels of *Bacteroides salyersiae* after FDR correction.

These contrasting results not only highlight the biological heterogeneity across disease states and anxiety subtypes, but also underscore the importance of rigorous statistical correction. A critical methodological consideration in microbiome research is the high dimensionality of microbial data and the large number of comparisons, which substantially increase the risk of false positives. Most previous studies did not apply FDR correction, which limits the reliability of their reported associations. In contrast, our study incorporated FDR correction, ensuring that the observed gut microbiota-anxiety associations in PD patients are more statistically robust and reliable. By controlling the false discovery rate at 5%, we reduce the likelihood of type I errors and enhance the credibility of our findings. This methodological rigor distinguishes our work from earlier studies and strengthens our contribution to the literature on microbiome-brain interactions in the context of Parkinson’s disease.

Our findings identified two bacterial genera that remained significantly associated with anxiety in PD after FDR correction. Specifically, an unclassified genus in the Ruminococcaceae family was enriched in anxious PD patients, while *Escherichia-Shigella* was less abundant in this group, indicating potential disease-specific microbial markers of anxiety in PD. This suggests that different bacterial taxa may contribute to the development of anxiety across distinct disease contexts. These findings align with MacKay et al. (2024), who proposed that different psychiatric disorders, such as generalized anxiety disorder and social anxiety disorder, may exhibit distinct microbiota-related mechanisms, reflecting disease-specific metabolic alterations.

Our functional analysis results suggest that the anxiety-related functional pathways may differ drastically between PD and non-PD individuals. In non-PD participants, anxiety appeared to be associated with glycosphingolipid and sphingolipid metabolism, pathways involved in cell membrane stability (Schengrund, 2015), synaptic function (Buccinnà et al., 2009), and neuroinflammation (De Wit et al., 2019). These predicted changes could potentially affect neurotransmitter signaling and contribute to anxiety. Additionally, putative alterations in glycan degradation could impact intestinal barrier integrity, potentially influencing gut-brain interactions (Dmytriv et al., 2024). In contrast, PD patients with anxiety were predicted to exhibit metabolic shifts in indole alkaloid biosynthesis and linoleic acid metabolism, which are linked to serotonin metabolism (Hamid et al., 2017) and neuroinflammation (Jantzen et al., 2024), respectively. Given that PD is characterized by serotonergic dysregulation and chronic inflammation, these pathways might play a distinct role in PD-related anxiety. Overall, our findings suggest potential differences in the functional mechanisms underlying anxiety, with non-PD anxiety possibly more related to neuronal membrane composition, while PD anxiety may be linked to disease-specific neuroinflammatory and metabolic alterations. Further confirmation of these functional predictions through direct metagenomic or metabolomic measurements would provide additional support to strengthen these findings.

This study has several limitations. First, the sample size was relatively limited, particularly in the anxious PD group. We acknowledge that the number of anxious individuals in the non-PD group was modest (N = 16), potentially limiting the ability to detect more subtle effects. These constraints may have contributed to false negatives, especially for microbial taxa or pathways with small effect sizes. Nevertheless, the consistent use of FDR correction throughout our analyses reduces the risk of false positives, lending greater confidence to the associations that remained statistically significant. Second, as a cross-sectional study, our findings cannot establish causal relationships between anxiety and gut microbiota alterations. Third, while we accounted for potential confounders such as age and sex, other environmental and genetic factors – including non-PD-related comorbidities and medications – were not fully explored. As information on these variables was not available, potential residual confounding due to polypharmacy could not be fully excluded. Fourth, anxiety symptoms were assessed using a self-reported questionnaire rather than formal clinical diagnoses based on ICD-10 or DSM-V criteria. As such, our classification reflects anxiety-related symptoms rather than clinically confirmed anxiety disorders, and may introduce non-differential misclassification bias. However, this type of bias would likely attenuate associations, making our significant findings conservative. Fifth, detailed clinical assessments of Parkinson’s disease severity - such as Hoehn and Yahr stage, UPDRS scores, or Levodopa Equivalent Daily Dose (LEDD) - were not available in our dataset, limiting our ability to fully characterize disease heterogeneity. Future research should incorporate larger, longitudinal studies to validate our findings and provide a clearer understanding of the dynamic interactions between gut microbiota and anxiety in PD.

This study demonstrates that the relationship between gut microbiota and anxiety differs between PD and non-PD populations, highlighting the gut microbiota’s potential role in PD-related anxiety. Future studies should explore causal mechanisms and assess microbiome-targeted interventions for anxiety management in PD.

## Data Availability

Publicly available datasets were analyzed in this study. This data can be found here: European Nucleotide Archive with the primary accession code ERP016332.
