# Supplementary material for: Anxiety-related gut microbiota alterations in Parkinson’s disease: distinct associations compared to healthy individuals
Source: Front Cell Infect Microbiol. 2025 Jun 18;15:1594152. doi: 10.3389/fcimb.2025.1594152 (PMC12213657; doi:10.3389/fcimb.2025.1594152)
Supplement: Supplementary file 1 [file Table1.docx]

**Supplementary materials of “Anxiety-Related Gut Microbiota Alterations in Parkinson’s Disease: Distinct Associations Compared to Healthy Individuals”**

Sheng-Hsuan Lin^1,2,3,4^, Ru-Jen Lin^5^, Kai-Yu Chan^4,6^, Chia-Ling Chu^1^, Yan-Lin Chen^1^, Shih-Chen Fu^4*^

**Table S1.** Demographic characteristics of participants with and without Parkinson’s disease.

|  | **Non-Parkinson's disease**  **(N = 129)** | **Parkinson's disease**  **(N = 193)** | **p-value** |
| --- | --- | --- | --- |
| **Anxiety** |  |  | 0.002 |
| No | 113 (87.6%) | 140 (72.5%) |  |
| Yes | 16 (12.4%) | 53 (27.5%) |  |
| **Gender** |  |  | <0.001 |
| Female | 52 (40.3%) | 129 (66.8%) |  |
| Male | 77 (59.7%) | 64 (33.2%) |  |
| **Age** |  |  | 0.085 |
| ≦ 65 | 36 (27.9%) | 73 (37.8%) |  |
| ＞ 65 | 93 (72.1%) | 120 (62.2%) |  |
| **Antibiotics** |  |  | 0.451 |
| No | 125 (96.9%) | 181 (93.8%) |  |
| Yes | 3 (2.3%) | 9 (4.7%) |  |
| Missing | 1 (0.8%) | 3 (1.6%) |  |
| **Probiotics** |  |  | 0.223 |
| No | 93 (72.1%) | 139 (72.0%) |  |
| Yes | 33 (25.6%) | 42 (21.8%) |  |
| Missing | 3 (2.3%) | 12 (6.2%) |  |
| **Eat fruits or vegetable daily** |  |  | 0.057 |
| No | 15 (11.6%) | 41 (21.2%) |  |
| Yes | 114 (88.4%) | 151 (78.2%) |  |
| Missing | 0 (0.0%) | 1 (0.5%) |  |
| **Eat grains daily** |  |  | 0.892 |
| No | 42 (32.6%) | 58 (30.1%) |  |
| Yes | 85 (65.9%) | 132 (68.4%) |  |
| Missing | 2 (1.6%) | 3 (1.6%) |  |
| **Eat meats daily** |  |  | 0.274 |
| No | 48 (37.2%) | 83 (43.0%) |  |
| Yes | 81 (62.8%) | 108 (56.0%) |  |
| Missing | 0 (0.0%) | 2 (1.0%) |  |
| **Eat nuts daily** |  |  | 0.366 |
| No | 92 (71.3%) | 151 (78.2%) |  |
| Yes | 36 (27.9%) | 41 (21.2%) |  |
| Missing | 1 (0.8%) | 1 (0.9%) |  |
| **Eat yogurt daily** |  |  | 0.603 |
| No | 115 (89.1%) | 176 (91.2%) |  |
| Yes | 12 (9.3%) | 16 (8.3%) |  |
| Missing | 2 (1.6%) | 1 (0.5%) |  |

**Table S2.** Additional demographic characteristics of participants with and without anxiety in the Parkinson’s disease (PD) and non-PD groups. These variables were not retained in the final model due to the absence of significant group differences. For participants without Parkinson’s disease, information on Levodopa use was not applicable and thus unavailable.

|  | **Non-Parkinson's disease** | | | **Parkinson's disease** | | |
| --- | --- | --- | --- | --- | --- | --- |
|  | **Anxiety (N = 16)** | **w/o Anxiety (N = 113)** | **p-value** | **Anxiety (N = 53)** | **w/o Anxiety (N = 140)** | **p-value** |
| **BMI** |  |  | 0.726 |  |  | 0.279 |
| mean (SD) | 28.823 (6.133) | 28.271 (5.669) |  | 27.176 (5.397) | 26.226 (5.316) |  |
| **Constipation** |  |  | 0.234 |  |  | 0.890 |
| No | 15 (93.8%) | 110 (97.3%) |  | 45 (84.9%) | 116 (82.9%) |  |
| Yes | 1 (6.2%) | 1 (0.9%) |  | 7 (13.2%) | 22 (15.7%) |  |
| Missing | 0 (0.0%) | 2 (1.8%) |  | 1 (1.9%) | 2 (1.4%) |  |
| **Levodopa** |  |  | - |  |  | 0.468 |
| No | - | - |  | 3 (5.7%) | 14 (10.0%) |  |
| Yes | - | - |  | 48 (90.6%) | 117 (83.6%) |  |
| Missing | - | - |  | 2 (3.8%) | 9 (6.4%) |  |


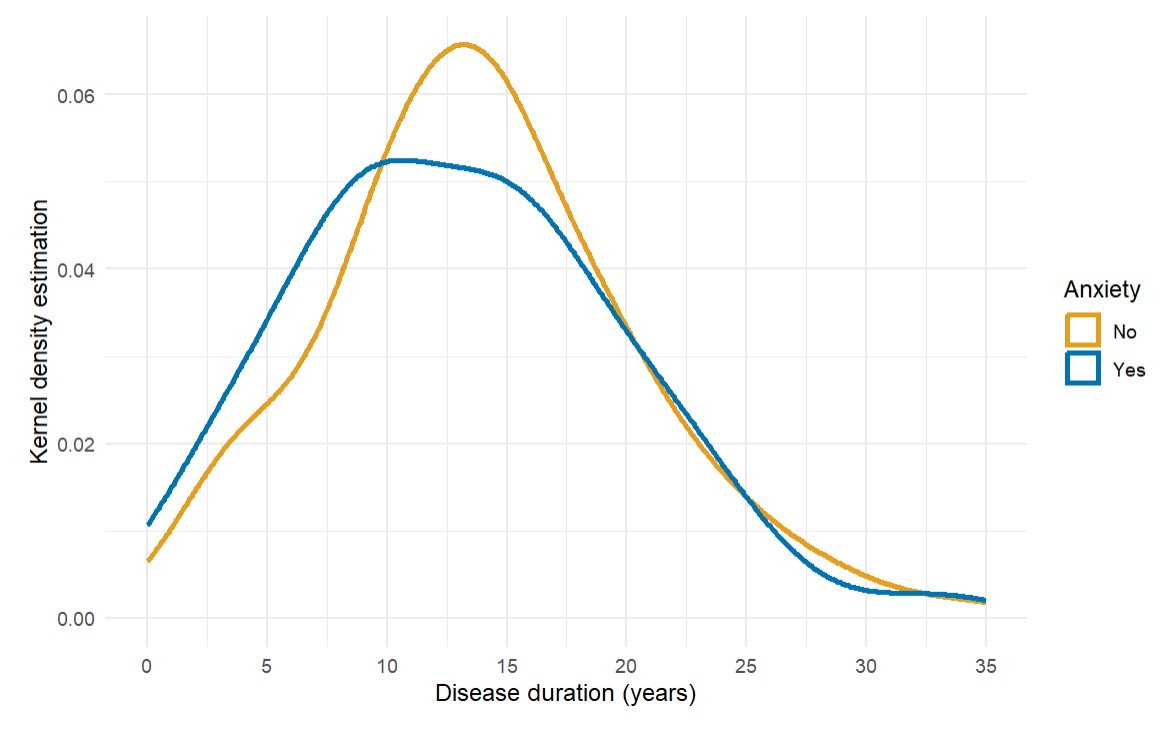


**Figure S1.** Kernel density estimation of Parkinson’s disease duration in patients with and without anxiety. This figure presents the smoothed distribution of disease duration among Parkinson’s disease patients, stratified by the presence or absence of anxiety. Kernel density estimation was performed using a Gaussian kernel, with the bandwidth scaled by a factor of 1.2. Although the density curves differ slightly in shape, the Kruskal–Wallis test indicated no statistically significant difference in disease duration between the two groups (p = 0.583).
